# The quality of selected raw and pasteurized honeys based on their sensory profiles and consumer preferences

**DOI:** 10.3389/fnut.2023.1330307

**Published:** 2024-01-16

**Authors:** Marek Kardas, Wiktoria Staśkiewicz-Bartecka, Katarzyna Sołtys, Lechowsław Dul, Anna-Maria Sapała, Agata Kiciak, Agnieszka Bielaszka, Justyna Kardas

**Affiliations:** ^1^Department of Food Technology and Quality Evaluation, Department of Dietetics, Faculty of Public Health in Bytom, Medical University of Silesia in Katowice, Zabrze, Poland; ^2^Department of Biostatistics, Faculty of Public Health in Bytom, Medical University of Silesia, Bytom, Poland; ^3^Doctoral School of the Medical University of Silesia in Katowice, Department of Human Nutrition, Faculty of Public Health in Bytom, The Medical University of Silesia in Katowice, Zabrze, Poland

**Keywords:** quantitative descriptive analysis (QDA), principal components analysis (PCA), honey, sensory quality, consumer preferences

## Abstract

The purpose of this study was to determine the sensory profile of honeys based on the method of quantitative descriptive analysis and principal component analysis and assess consumer preferences of raw and pasteurized honeys. Samples of multi-floral honeys (from the store and apiary) were subjected to sensory analysis based on the method of ranking for taste preference, the method of scaling based on color, aroma, taste, and texture, and the method of differential descriptive analysis using 11 quality descriptors. The results were subjected to statistical analysis using the Principal Component Analysis method. The taste was found to be a descriptor that differentiates honey by origin. Consumers prefer the taste of pasteurized honeys. As a result of assessing the quality of honeys using the scaling method, it was found that: raw honeys are characterized by a lighter color than pasteurized honeys, store-bought honeys have a less noticeable aroma than honeys obtained from beekeepers, while samples of pasteurized honeys were judged to have a consistency more like that of typical honey. The sensory profiles obtained highlight the differences between pasteurized honeys and raw honeys.

## 1 Introduction

Due to its composition and properties, honey is one of the most valuable animal products used by humans (1). It is a naturally sweet substance produced by bees, it is synthesized from the nectar of flowers, from the excretions of living parts of plants, or from the secretions of insects sucking the juices of living parts of plants, which bees collect, carry, and combine with specific substances of their own, deposit and leave to mature in honeycombs (2, 3). This substance is known as a food product, due to its flavorful qualities it has applications in culinary technology: for spreading bread, as a sweetener, as the base of sweet sauces, and as an ingredient in various desserts. On the other hand, honey is also used as a remedy for many ailments; herbal drinks, ointments, and medicinal patches are made from it (1, 4, 5). It is valued for its multidirectional properties. The components of honey have bactericidal and bacteriostatic effects. In addition to its nutritional value, it also has phytochemical, anti-inflammatory, antimicrobial, and antioxidant effects (6). The factors that provide the antibiotic effect of honey can be divided into three main groups: physical, such as high osmotic pressure and acidic reaction; enzymatic, such as the content of glucose oxidase and lysozyme; and chemicals, such as the content of essential oils, flavonoids, organic acids, and tannins (5). Protein and enzymatic factors have antibiotic properties. An important substance is glucose oxidase, which in honey in the course of a chemical reaction leads to the formation of gluconolactone, which combined with water gives gluconic acid and hydrogen peroxide, with antibacterial and antifungal activity (5, 7–9). Another compound that affects antibiotic activity is lysozyme, a small-molecule, a thermostable protein that is responsible for the phenomenon of lysis of the cell walls of Gram-positive bacteria (10, 11). There are also other thermostable substances in honey, such as essential oils, flavonoids, and tannins. Storing honey under unsuitable conditions and heating it to temperatures above 45°C causes it to lose its enzymatic properties (8, 10). Honey has strong antioxidant properties, for which enzymes, vitamins, and polyphenols are responsible, whose content ranges from 0.01 to tens of mg/kg (12–14). Thanks to all these properties, honey is used in cosmetics, the production of medicines, as well as in the prevention of diseases. Its multidirectional use in the treatment of diseases is so important that it has received its own name apitherapy. Ancient civilizations considered honey a gift, so its importance is described in all religions. (5, 15–17). Thanks to the development of science, there are various means to evaluate the composition and biological and therapeutic properties of honey. There are different types of honey, which differ in composition, physicochemical properties, and biological effects (18).

Based on its origin, honey is divided into nectar, honeydew, and nectar-honeydew. In addition, the Polish Standard also distinguishes varieties of nectar honey, such as acacia, lime, buckwheat, heather, and multi-flower (19).

Nectar honey is produced by bees from the nectar of plants. Honeydew honey is honey made mainly from the excretions of insects sucking the juices of living plant parts or their secretions. On the other hand, nectar-honeydew honey is a mixture of these two varieties (19).

Color, flavor, aroma, and texture are features that characterize the different varieties of honey and make it possible to differentiate them (19). The peculiar organoleptic properties of honey are determined primarily by the type and species of the plant from which the pollen was collected. Among honeys, a wide variety is observed in terms of color. Individual varieties of honey can range in color from light cream to brown (19, 20). The color of honey is determined by factors such as the type of forage, environmental factors (temperature, soil, humidity), and storage time (19, 21). Honey color is also influenced by the content of natural pigments: carotenoid compounds, xanthophyll, chlorophyll and its derivatives, flavonoids, and anthocyanins (5, 20). The different varieties differ significantly in palatability. The main component determining the flavor of honey is sugars; those with a higher fructose content than glucose are considered sweeter (15, 16, 22). Other compounds that determine the taste of a particular variety of honey are organic acids (gluconic acid, citric acid, malic acid, acetic acid), tannins, glycosides, and alkaloidal substances (5). The consistency of honey depends on the advancement of the crystallization process. Honey can have a liquid, viscous, partially or completely crystallized consistency. The storage temperature of the product has a significant effect on the crystallization process of honey and consistency (5, 15).

Today, interest in honey is very high, as there is a growing interest in natural medicine, healthy eating, and taking care of appearance. However, it is important to distinguish raw honey, which, through the absence of technological processes, is a product with greater biological value than honey subjected to pasteurization.

The sensory qualities of a product play an important role in its acceptance. This is because consumers do not want to buy and consume products that do not meet their expectations. Evaluation of consumer preferences is very important and valuable information for food manufacturers, but it is not enough. After all, a producer needs to know not only how his product is rated by consumers, but also why it received a certain rating. Quantitative descriptive analysis (QDA) provides valuable answers to this question.

The purpose of this study was to determine the sensory profile of honeys based on the method of quantitative descriptive analysis, and principal component analysis, and to assess consumer preferences and the quality of raw and pasteurized honeys.

## 2 Materials and methods

### 2.1 Sample collection

The study material consisted of 16 samples of nectar, multifloral honeys of various origins obtained in the second quarter of 2022. The honey samples were divided into two categories. The first category consisted of samples of raw, unprocessed honey obtained from beekeepers from two apiaries located in Poland, in Silesia Province (Raw Samples). The honeys were classified as spring honeys, which were produced from spring-flowering plants in various proportions: lime, turnip, sunflower, buckwheat. All honey samples were not heated by producers and were taken no later than 4 weeks after extraction from the hives, the batches showed no signs of fermentation or crystallization. The honey samples were stored at room temperature until use. Both apiaries used bees of one subspecies, *Apis mellifera carnica*. The second category consisted of nectar, multifloral, pasteurized honeys purchased from two shopping centers in the city of Katowice (Silesia Province, Poland), these honeys were a mixture of honeys from EU and non-EU member states (Pasteurized Samples). Honey producers did not declare detailed information on the time of harvesting honey and the species of bees. According to the producers, the honeys were pasteurized, heated at 80°C for 3 min, then immediately cooled to 45°C. Four samples were taken from each sample acquisition point, which were then mixed to obtain a representative sample for further analysis before proceeding. Samples were combined under laboratory conditions at 21 degrees C with full sterility of methods.The samples of multifloral honeys used in the study were coded and labeled with symbols (according to the place of origin): Raw Sample 1, Raw Sample 2, Pasteurized Sample 1 and Pasteurized Sample 2.

The honeys were stored in a dry, dark, and cool place. Prior to sensory testing, honey was recrystallized and samples of one origin were combined in a water bath at 40°C ± 1°C to standardize consistency.

### 2.2 Study design

Appropriately coded and prepared samples were subjected to sensory analysis using 3 methods. The research was carried out in the sensory laboratory of the Department of Dietetics of the Department of Food Technology and Quality Evaluation, Medical University of Silesia, designed in accordance with the standard: PN-EN ISO 8589: 2010 (23). The laboratory had 6 separate workstations, providing the assessors with appropriate conditions to conduct the test (elimination of disturbing factors; noise, bright light, etc.). The samples for evaluation were administered in odorless, colorless, plastic containers intended for contact with food.

The study was conducted with the approval of the Bioethics Committee of the Silesian Medical University in Katowice (KNW/0022/KB/16–1/14).

### 2.3 Evaluation by serialization method

In the first stage of the research, the honey samples were assessed by the serialization method, using the author’s assessment card. The samples had to be ranked from most to least palatable by writing the sample codes in the correct order. The evaluation group consisted of 75 people (36 women and 29 men) trained in the scheduling method. The evaluators ranged in age from 18 to 30, with an average age of 23.43 ± 2,43.

### 2.4 Evaluation by scaling method

In the second stage, the honey samples were assessed by the scaling method, using the proprietary five-point scale, assessing the samples on the basis of four differentiators: color, smell, taste, and consistency. The evaluators were asked to evaluate the samples using a 5-point hedonic scale. The boundary values of the scale are shown in the table below (Table 1). The values from 2 to 4 corresponded to the intermediate intensity of the examined feature.

**Table 1:** Border markings of the 5-point scale.

Sample characteristic	1	5
Color	Bright, cream-colored	Dark, pale yellow color
Smell	Impalpable	Very palpable
Taste	Impalpable	Very palpable
Texture	Adequate for honey	Inadequate for honey

The evaluation group consisted of people who participated in the serialization method and were properly trained for the described evaluation.

### 2.5 Quantitative descriptive analysis

The last method was the quantitative descriptive analysis (QDA) in accordance with the executive procedure described in ISO 13299:2016–05 (24). To conduct the study, an original questionnaire was used, for the preparation of which the qualitative attributes for the evaluation of honey were selected and defined. The study used 10 descriptors for odor, taste, smoothness, viscosity, and mouthfeel. The descriptors are summarized in Table 2.

**Table 2:** Discriminators used in the QDA assessment.

Characteristic	Definition
Color	Typical for honey: from bright, cream-colored to dar, pale yellow colour.
Smell of beeswax	The smell is characteristic of beeswax.
Sweet-nectar smell	Mild, sweet, nectar-like smell.
Sweet taste	The basic quality of a sweet taste does not need to be defined.
Honey taste	The taste is characteristic of honey.
Burning-irritating taste	A slight burning sensation in the mouth and throat, irritation of the taste buds.
Acerbic taste	A tightening sensation, especially felt at the edges of the tongue and the walls of the mouth.
Foreign taste	Taste to stray from the typical honey taste
Smoothness	Uniform structure of the sample when spreading it through the mouth.
Stickiness	A feeling of sticking the surface of the tongue to the top of the mouth.
Dissolving in the mouth	A feeling that the sample is melting in the mouth.

For the quantification of the descriptors, a linear scale with the given boundary terms was used. The intensity of the descriptors assessed increased from left to right in order to minimize the possibility of error.

The sensory characteristics of the samples were performed by a 10-person evaluation team (in two independent repetitions), properly trained, and prepared methodically in accordance with the PN-ISO 4121: 1998 (25) and PN-ISO 6564: 1999 standards (26).

### 2.6 Statistical analysis

The obtained data were developed using Statistica v.13.3 (Stat Soft Polska) and R v. 225 4.0.0 package (2020) under the GNU GPL license (The R Foundation for Statistical Com-226 puting).

To present quantitative data, mean values, and standard deviations were calculated - X ± S. For qualitative data, percentage notation was used. Qualitative data were expressed as numerical values determined by mathematical methods to make statistical inferences. The Friedeman test (the non-parametric equivalent of one-way analysis of variance) was used to analyze the variation in rank sums. In order to check the compliance of the assessments of tested products, the W Kendall coefficient of conformity (WK) and the Spearman similarity coefficient (rS) was used. Kendall’s W coefficient of concordance is the arithmetic mean of Spearman’s rank correlation coefficients calculated for each pair of subjects evaluating the samples analyzed. Spearman’s similarity coefficient was used to test the correlation between the ratings (more precisely, the ranks) given to each sample by the respondents. The more the ordering of the honey samples differed for a pair of subjects, the closer rS was to 0. Kendall’s W concordance coefficient takes values between 0 and 1, where 0 means there is a complete lack of concordance between the ratings assigned to the honey samples by the subjects, while 1 means that all subjects assigned the same ranks to the honey samples.

Principal Component Analysis (PCA) is a method used to interpret sensory profiling results. The extensive use of PCA in QDA is based on the concept of a spatial n-dimensional model of a product’s sensory quality (or its single modality, such as aroma, texture, or palatability) determined by a set of discriminants, or descriptors.

Principal Component Analysis makes it possible to study a large number of data, taking into account the interdependencies between them. The results of PCA are presented in graphical form (the so-called PCA projection of differences and similarities in sensory quality of the food products studied). In Principal Component Analysis, a coordinate system was created, the axes of which are the so-called principal components, and by analyzing the correlation between the primary variables (i.e., the values of the relevant differentiators for the tested honey samples) and the obtained principal components, the primary variables (i.e., the differentiators, descriptors describing the tested products) were reduced and the differentiators and the tested products were clustered.

A value of *p* < 0.05 was used as a criterion for statistical significance.

## 3 Results

The analysis of the results from the honey sample analysis by the ranking method was started by summing up the ranking positions of individual samples on each evaluation sheet. A scaling method was also used based on four discriminants: color, smell, taste, and texture. After assigning each of the ratings a numerical value and summing them up by counting the ranks, the results presented in Table 3 were obtained.

**Table 3:** Assessment of the intensity of the examined honey features (rank sums) by the ranking method (*N* = 75).

Honey sample	RANK SUMS				
	Color	Smell	Taste	Consistency	General
Raw Sample 1	152^b^	240^b^	152^b^	258^b^	214^b^
Pasteurized Sample 1	271^a^	175^a^	236^a^	149^a^	142^a^
Raw Sample 2	108^b^	283^b^	244^c^	264^b^	212^b^
Pasteurized Sample 2	309^c^	142^a^	208^a^	169^a^	182^a^

The values of the sum of the ranks within the ratings of the individual distinguishing factors marked with different letters are statistically significantly different (*p* < 0.05).

The values of the sum of ranks within the ratings of individual discriminants marked with different letters significantly differ statistically.

**Figure 1:**
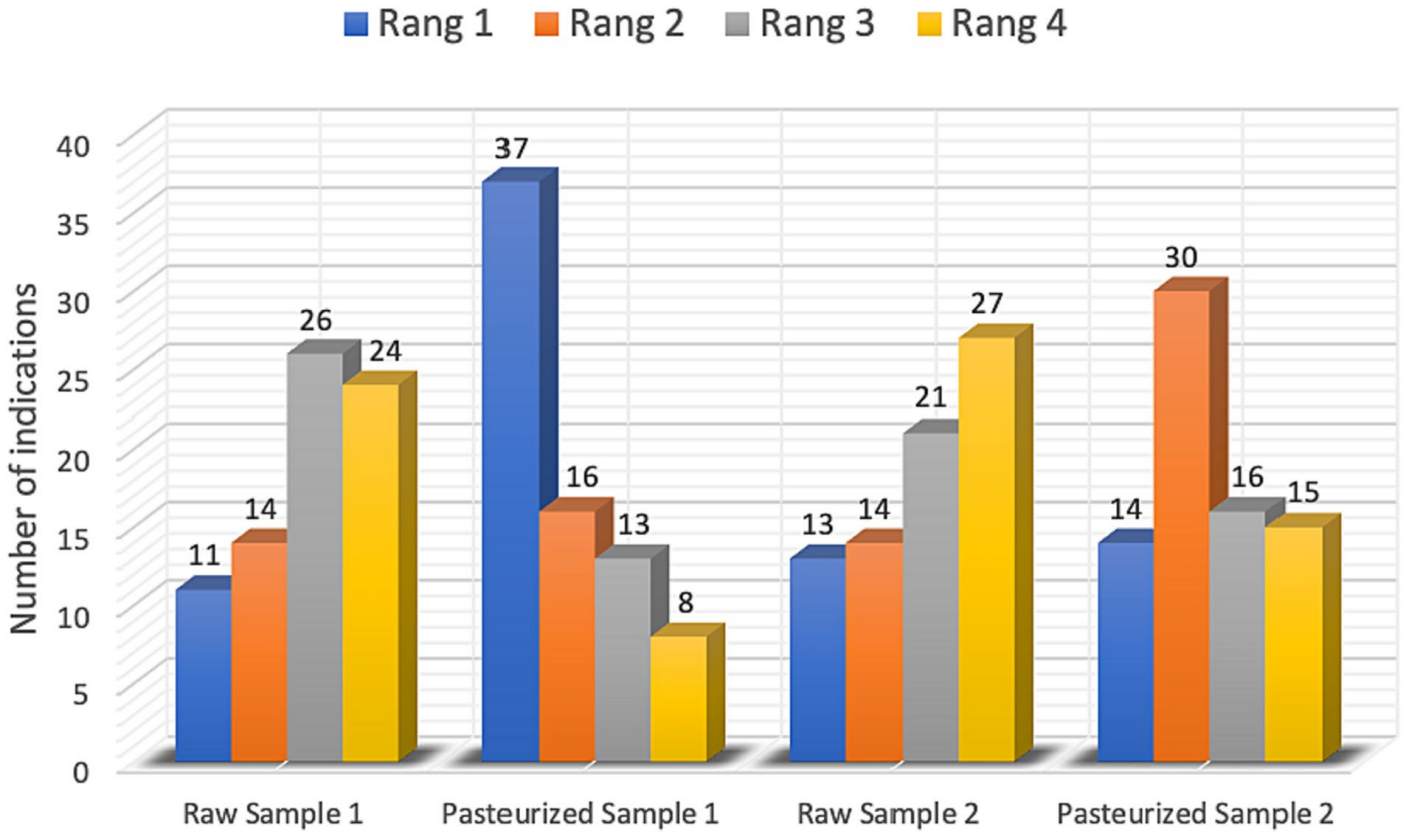
Number of indications in the serialization survey. Rang 1 rated as “the best” to rang 4 classified as “the worst,” according to the researchers’ assessment.

On the basis of the obtained sums of ranks, it was found that the pasteurized honeys were rated as tastier than the raw honeys. According to the evaluators, the Pasteurized Sample 1 turned out to be the tastiest sample, while the Raw Sample 1 was the least tasty. In order to assess the agreement of the respondents’ ranking of the samples of honey analyzed, Kendall’s W coefficient of agreement (WK = 0.12) and Spearman’s similarity coefficient (rS = 0.11) were calculated. The obtained values indicate very low agreement of consumers’ ratings of the analyzed honey samples. Details of the ranks awarded are shown in Figure 1.

The results indicate that raw honeys are much lighter. According to the evaluators, the lightest sample was Raw Sample 2, then Raw Sample 1, Pasteurized Sample 1, and the darkest one - Pasteurized Sample 2, there was good agreement in the rater responses. Based on the calculated Kendall’s W coefficient of concordance (WK =0.84) and similarity coefficient (rS = 0.84), it can be concluded that the concordance of the evaluators’ responses was high. The statement that the color of the samples depends on their origin is plausible.

Based on the results, differences can be seen between the smells of pasteurized honeys and those obtained from apiaries. As the sample with the least noticeable odor, the evaluators considered the sample Pasteurized Sample 2, followed by Pasteurized Sample 1, Raw Sample 1, and Raw Sample 2. The calculated coefficient of concordance W Kendall (WK = 0.39) and the coefficient of similarity (rS =0.39) indicate low concordance of the evaluators’ answers.

As the sample with the most intense taste, the evaluators selected the sample of Raw Sample 2, then Pasteurized Sample 1 and Pasteurized Sample 2. The sample of Raw Sample 1 was selected as the sample with the least palpable taste. As a result of the analysis of the obtained data, the Kendall W concordance value (WK = 0.18) and the coefficient of similarity (rS = 0.17) were obtained. The obtained values indicate a low agreement of the evaluators’ answers, although higher than with the scheduling method for the same discriminant.

**Figure 2:**
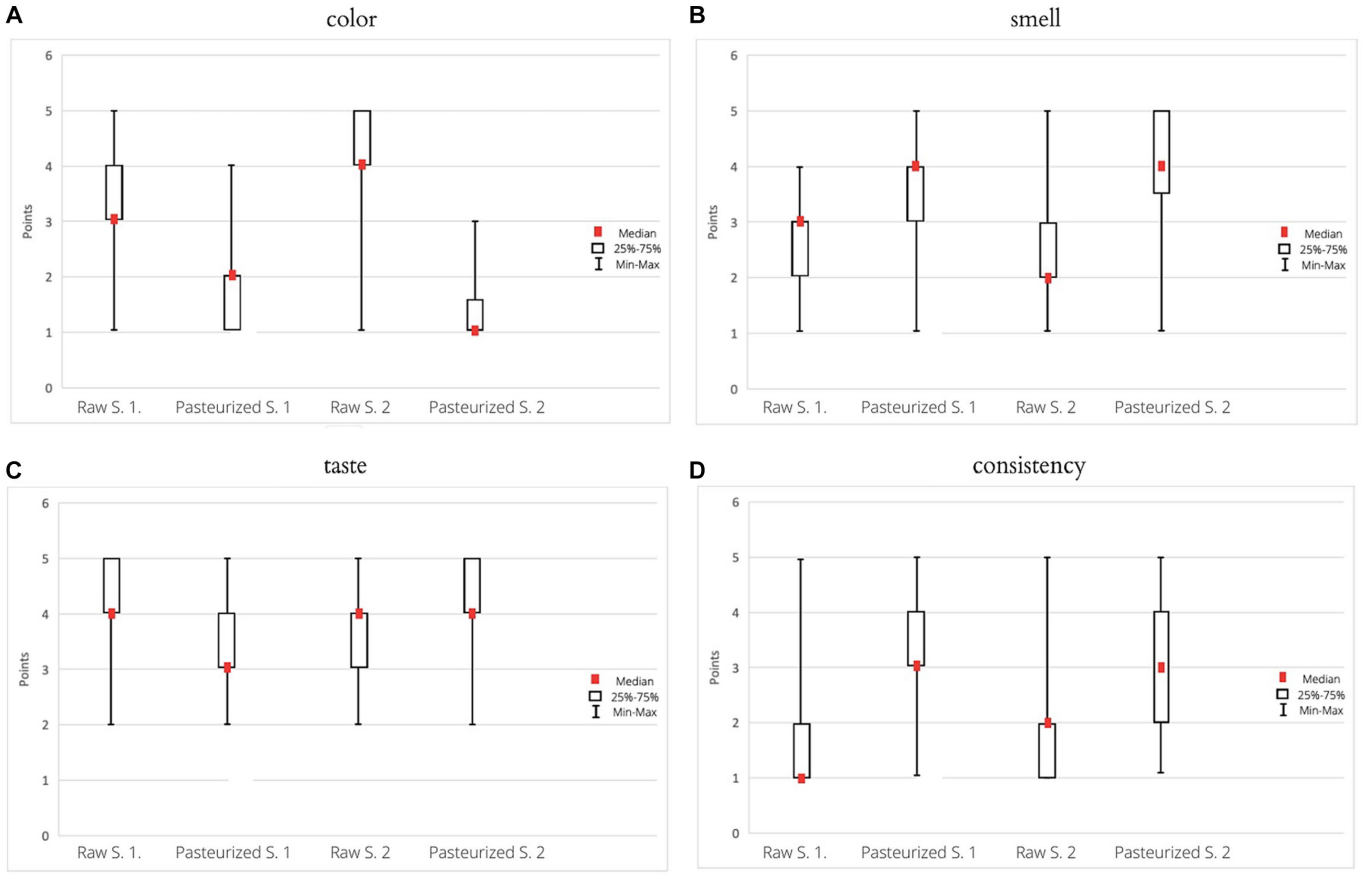
Results of evaluation of the intensity of the color **(A)**, smell **(B)**, taste **(C)**, and consistency **(D)**.

As the sample with the consistency most similar to that suitable for honey, the evaluators indicated the sample Pasteurized Sample 1, then Pasteurized Sample 2, Raw Sample 1, and Raw Sample 2. Analyzing the above data, the following values were obtained: WK = 0.35 and rS = 0.34, which indicates a low concordance of the evaluators’ answers. Figure 2 presents the obtained results graphically.

As a result of the evaluation of the honeys, in duplicate, using the QDA method, the mean scores were obtained, which are summarized in Table 4.

**Table 4:** QDA ratings of honeys with standard deviation in two replicates.

Sample	Raw 1 X ± SD	Raw 2 X ± SD	Pasteurized 1 X ± SD	Pasteurized 2 X ± SD
C	4.90 ± 0,3	6.15 ± 0.25	8.00 ± 0.14	8.30 ± 0.00
SB	5.00 ± 0,3	7.25 ± 0.07	6.85 ± 0.07	5.80 ± 0.28
SNS	4.45 ± 0.35	8.20 ± 0.14	6.90 ± 0.28	6.40 ± 0.28
ST	8.70 ± 0.14	9.35 ± 0.07	8.30 ± 0.28	9.20 ± 0.14
HT	7.35 ± 0.21	9.25 ± 0.07	6.65 ± 0.07	6.40 ± 0.21
BIT	3.70 ± 0.14	1.55 ± 0.35	1.90 ± 0.00	0.85 ± 0.05
AT	2.30 ± 0.14	1.55 ± 0.07	1.65 ± 0.21	1.30 ± 0.00
FT	1.10 ± 0.28	0.65 ± 0.07	2.40 ± 0.43	1.80 ± 0.14
SM	6.20 ± 0.00	6.40 ± 0.07	8.40 ± 0.43	7.80 ± 0.14
S	6.75 ± 0.21	8.85 ± 0.07	8.70 ± 0.00	8.50 ± 0.28
DM	7.70 ± 0.00	8.50 ± 0.42	8.25 ± 0.64	8.60 ± 0.42
G	7.75 ± 0.21	8.50 ± 0.42	7.25 ± 0.21	7.25 ± 0.21

C – color, SB – the smell of beeswax, SNS – sweet-nectar smell, ST – sweet taste, HT – honey taste, BIT – burning-irritating taste, AT – acerbic taste, FT – foreign taste, SM – smoothness, S – stickiness, DM – dissolving in the mouth, G – general.

**Figure 3:**
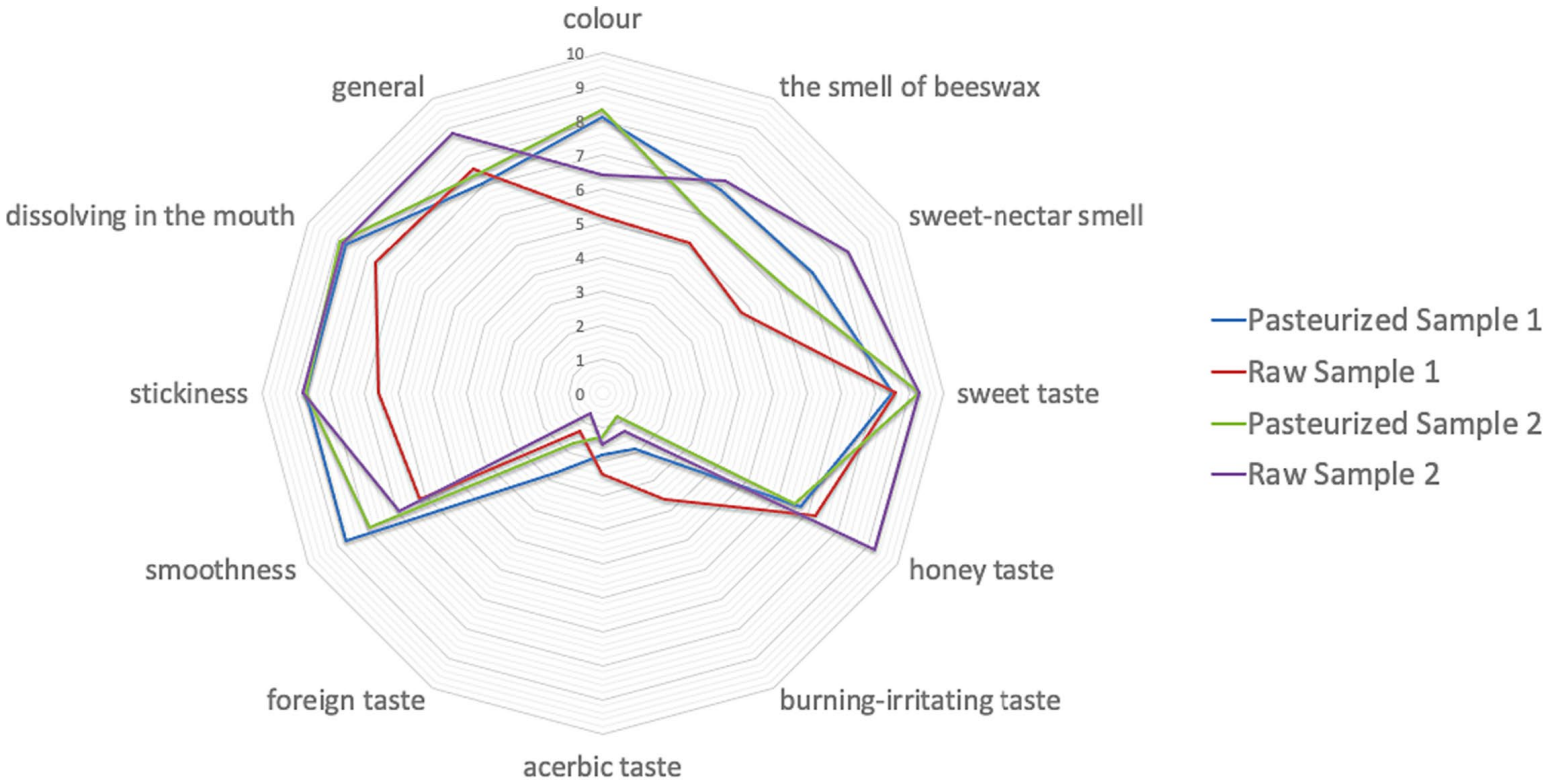
Radar plot showing the intensity of individual descriptors in duplicate QDA tests.

On the basis of the averages obtained, the intensity of individual descriptors was presented graphically (Figure 3).

**Figure 4:**
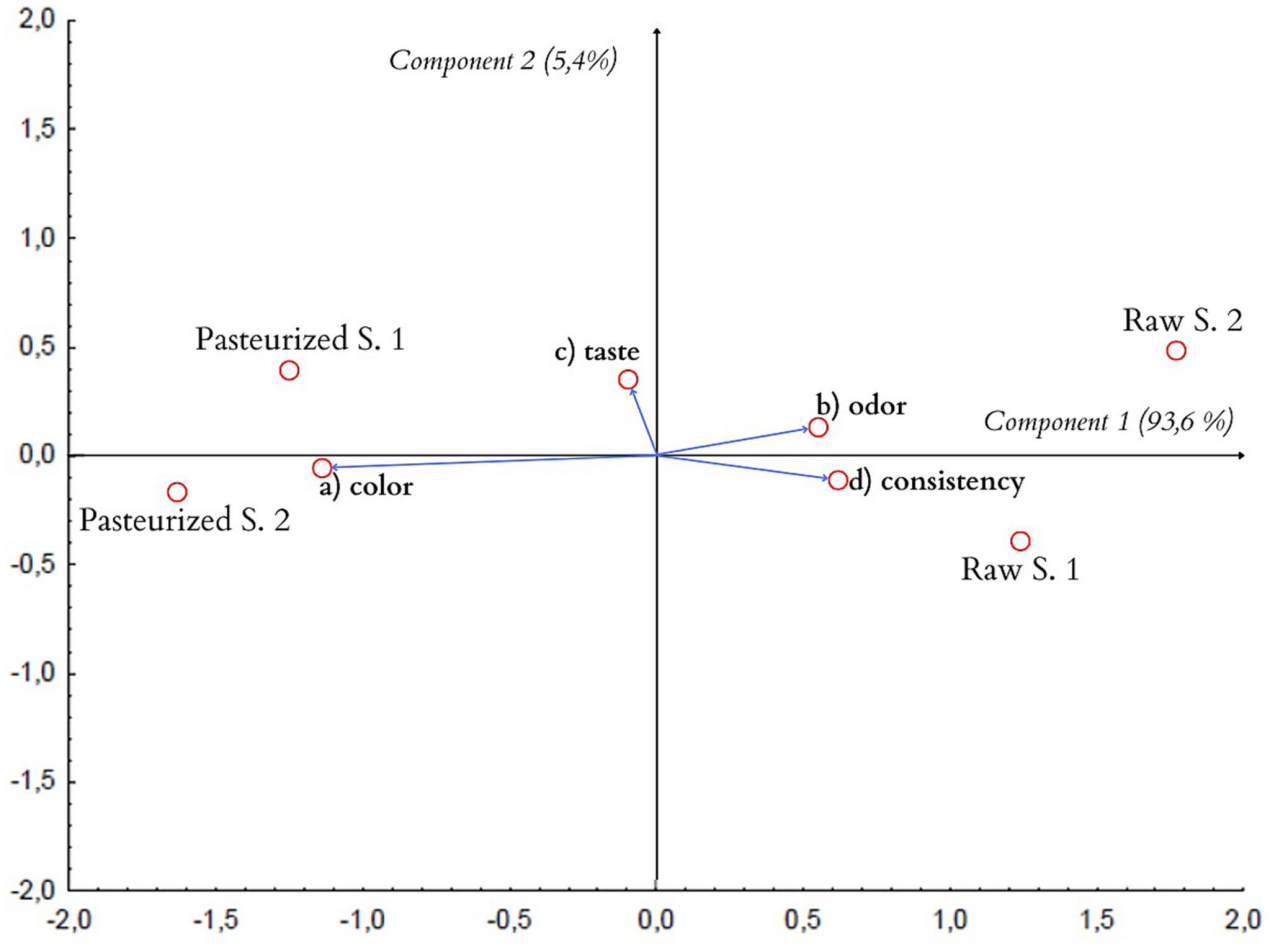
PCA projection of similarities and differences in sensory quality of the tested honeys (biplot).

The data obtained as a result of tests of honey samples carried out by the scaling method and the QDA method were analyzed by the PCA principal components method. The results of the analysis are presented in Figure 4.

Based on the PCA analysis, the honey samples were classified based on two factors explaining the variability of the original data. In a study by respondents evaluating honey samples using the scaling method, component 1 explains 93.6%, while component 2 explains 5.4% of the variability of all the discriminants used in the study.

As can be seen from the graphic PCA projection of the similarities and differences in the sensory quality of the samples of the tested honeys, two groups of samples of similar sensory quality were created. The groups discussed are located in different halves of the graph, which correspond to the different signs of the factor axis.

The length of the vector, which corresponds to the “taste” descriptor, in relation to component 1 is very small, which means that this feature has a very weak effect on the differentiation of both examined groups of honeys. Pasteurized honey samples clearly differ in color from samples of raw honeys. On the other hand, honey samples from apiaries have a more intense smell and consistency compared to honey samples from stores.

Subjecting the data obtained by QDA tests were subjected to statistical analysis using the PCA method, on this basis, the classification of the tested honey samples was made based on 2 factors explaining the variability of descriptors.

**Figure 5:**
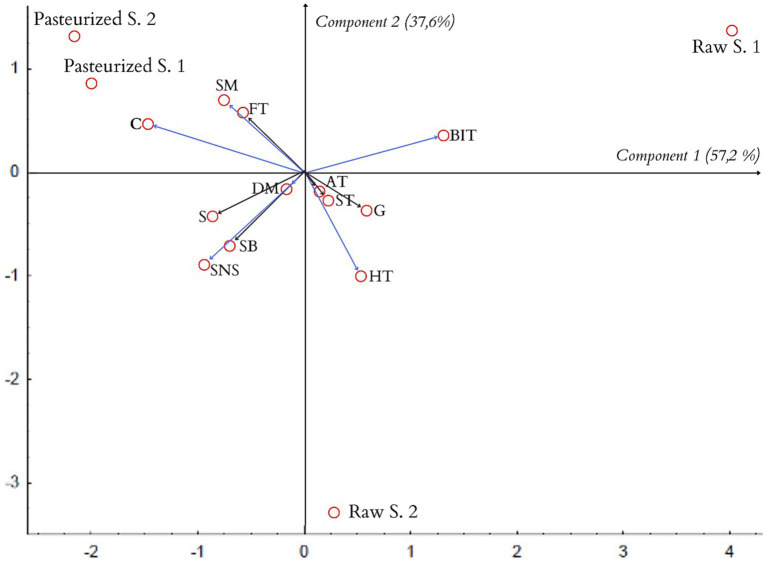
PCA projection of the similarities and differences in sensory quality of the tested honeys in the first study (biplot).

In the first study, component 1 explains 57.2%, while component 2 explains 37.6%, while in the second repetition, component 1 explains 57.8%, and component 2 explains 35% of the variability of all discriminants used in the study (Figure 5).

The analysis of the results from the first repetition of the QDA test shows the presence of three groups of samples of the tested honeys: Pasteurized 1 and Pasteurized 2, Raw 1, and Raw 2.

Pasteurized honeys samples are characterized by a high similarity in their sensory quality. Color and smoothness are the descriptors that clearly distinguish the pasteurized-samples of honey from raw-samples of honey (27).

The sample of Raw 1 honey has a more intense, burning-irritating taste compared to the other samples. On the other hand, the honey sample of Raw 2 is characterized by a more intense honey flavor and a sweet-nectar aroma compared to other samples.

**Figure 6:**
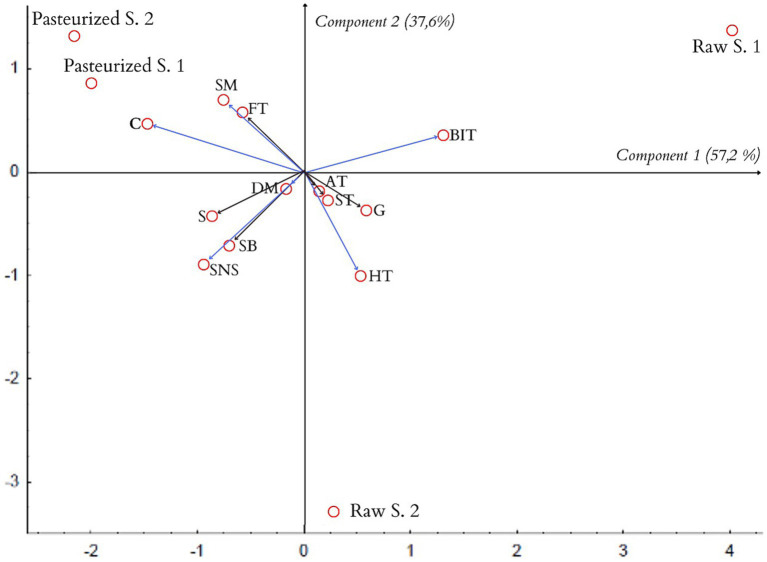
PCA projection of the similarities and differences in sensory quality of the tested honeys in the second study (biplot).

The results of the PCA statistical analysis of the results obtained in the second replication of the QDA study are presented in Figure 6.

The results of the repeated tests, presented on the graphic PCA projection of the similarities and differences in sensory quality of the samples of the tested honeys (Figure 6), confirm the conclusions obtained in the first study.

## 4 Discussion

All the honeys tested were multiflorous honeys. Nevertheless, differences in the assessment of the tested honey sensory features and the perception of individual descriptors were visible. This may be due to differences in the botanical regions of the samples. Multi flower honeys can differ significantly from one another depending on the origin of the nectar collected and used by bees.

The apiaries from which the honey was obtained, Raw Sample 1 and Raw Sample 2, were located in the Silesian Province. The labels honeys’ samples Pasteurized 1 and Pasteurized 2 contained the information - “A mixture of honeys from EU and non-EU Member States.,” Which indicates that the exact origin of the honey is unknown. The composition of honey blends can be very diverse. The information on the label that the product is such a mixture is a gateway for producers to market honey of unknown origin, without adequate information for consumers.

The research on preferences regarding the choice of the place of honey supply shows that buyers prefer honeys from private apiaries. Aldona Gontarz et al., analyzing the consumer preferences of students regarding honey, showed that 67.1% of respondents prefer to buy honey directly from the producer (28). Similar conclusions were obtained on the basis of research carried out by Bratkowski et al., in which as many as 84.8% declared that they most willingly obtain honey directly at the beekeeper’s house (29). This form of selling honey allows the customer to contact the beekeeper directly. The consumer has the opportunity to obtain the necessary information which increases the awareness of the buyer.

The consumer assessment using the ranking method showed differences in the preferences of respondents regarding the taste of honey. Honey from shopping malls was rated as tastier. Comparing the assessment of taste preferences in the conducted study to the questionnaire research on the declared preferences regarding the place of honey purchase, it can be concluded that it is contradictory. This is due to the fact that the samples were encoded and the place of origin was unknown to the respondents.

As a result of the evaluation of the color intensity of the tested samples, raw honeys were considered darker. Color variation within one variety may depend on the degree of consistency. Pasteurized honeys were poured, while the consistency of the honeys from the apiaries was a bit dense. Differences in color may also result from differences in the type of fruit used in the production of honey and the period of its production. Raw honey obtained from apiaries turned out to be sampled with a more intense smell. The results indicate that the evaluators noticed significant differences in the perception of smell between the tested honey samples.

Sensory evaluation, next to chemical, physical, and microbiological tests, is an important element of all kinds of food analysis and evaluation. The scheduling method requires the evaluator to rank the samples according to appropriate guidance. It is not possible to evaluate two samples equally. In the study, the evaluators had to rank the honey samples from the most delicious to the least tasty according to their preferences. This method allowed for greater differentiation between the assessed samples than in the scaling method, where respondents could assign the same number of points to a different sample of honey.

The data obtained as a result of the scaling methods were analyzed with the PCA method. The charts included in the work, obtained as a result of the PCA analysis, have the form of a “qualitative map.” They allow to assess the similarities of the sensory quality of the tested honey samples on the basis of their location in relation to each other in the system of main components - the closer the honey samples are, the greater the similarity of their sensory quality. The distance between the samples of the tested honeys on the PCA projection allows for assessing the differences between the samples.

Creating a biplot of the analyzed descriptors allows for a fairly simple indication of which descriptors and to what extent the examined honey samples differentiate. Based on the vectors created in the system of principal components, which begin at the beginning of the coordinate system and end at the point of the analyzed discriminant, it is possible to assess the degree of differentiation of the honey samples - the greater length of the vector means that the given descriptor differentiates the examined honey samples more strongly. The distribution of honey samples on the biplot diagram, concerning the QDA method used in the first repetition, indicates the division of the tested samples into groups. This division suggests a sensory similarity of pasteurized honeys from stores. Also, the results for the sensory evaluation at the consumer level indicated that the “color” discriminant clearly distinguished the tested samples of pasteurized honeys from samples of raw honeys.

The obtained graphs (Figures 4, 5) show that the conclusions obtained on the basis of the scaling method concerning the color intensity coincide. They show that the color of pasteurized honeys is the feature that distinguishes them most from the other samples.

Raw honey samples were smooth. In the scaling method, the respondents indicated the above samples as having a consistency more suitable for honey. In consumer research on the preferences of bee honeys (30, 31), as many as 48.78% of respondents declared that they prefer liquid honeys.

The QDA method used in the study characterizes the analyzed samples in detail. It is used for sensory analysis of bee honey, which is considered an important tool for determining its floral origin. Ciappini et al. (32) tested samples of Argentine honeys using QDA. In order to determine the sensory profile, clover and eucalyptus honey were assessed. Significant differences in perceived descriptors were shown. Clover honey was characterized by a fruity and floral taste of low intensity, while eucalyptus honey had a more intense flavor, with a floral note and an aromatic scent (30, 31, 33).

Indian honeys were also analyzed using the QDA method (32). The study included 11 samples of multiflorous honeys, which were grouped using PCA according to sensory variables and physicochemical parameters in order to analyze the relationship between the groups. The usefulness of the QDA method in the sensory tests of honey in combination with the use of physicochemical tests can be used to distinguish the floral origin and the fruit used by bees in order to differentiate the tested samples, as well as to characterize the individual varieties of honey.

Where honey is bought is an important factor in determining the quality of honey and its health-promoting qualities. In recent years, a number of producers have appeared in the retail chain, buying honey from apiaries, packaging it, and selling it. Such honey requires careful veterinary inspection for quality, as it is one of the food products that are often adulterated, and it is almost impossible for the consumer to distinguish a quality product from an imitation on his own. Adulterated honey is characterized by a lower content of compounds that have a positive effect on human health (34, 35).

The study by Kędzierska-Matysek et al. analyzed the mineral content of honeys purchased from apiaries and stores available on the Polish market. Honeys purchased directly from the producer contained more K, Mg, and Mn (36). A study by Balzan et al. analyzed the microbiota of honeys, it can provide information on production in a contaminated environment and failure to follow good beekeeping and production practices, which may be less dangerous in terms of food safety and affect product quality. Honeys from market sales presented inferior quality. Honeys obtained from beekeepers are characterized by very low amounts of contaminating microorganisms, and beneficial spore-forming bacteria may be present (37).

There are some limitations of the current study. The samples analyzed came from 2 apiaries and 2 stores, it would be reasonable to increase the number of places from which honey samples were obtained. It would also be worth considering expanding the study to include chemical analysis of the samples and comparing it with consumer preferences. On the other hand, an important value of this work is to obtain practical results that can provide important information for beekeepers about the preferred characteristics of this product. Obtaining honey with desirable characteristics by beekeepers can increase the interest of potential consumers, this phenomenon is also important with regard to the possible health-promoting effects on the health of honey obtained from apiaries.

## 5 Conclusion

Based on the results, it was concluded that the analysis of sensory characteristics of honey samples showed significant differences between honeys taken from different points. Taste is a descriptor that differentiates honey in terms of its origin. Consumers prefer the taste of pasteurized honeys purchased in stores to those raw from apiaries. In addition, raw honeys are characterized by a lighter color than pasteurized honeys, store honeys have a less noticeable aroma than honeys obtained from beekeepers, and samples of pasteurized honeys according to respondents have a texture more suitable for honey. The sensory profiles obtained highlight the differences between pasteurized honeys and raw honeys. Honeys from stores are characterized by high similarity in sensory quality. Distinguishing characteristics of raw honeys obtained from apiaries include honey taste, burning-irritating taste, and sweet-nectar aroma. Obtaining a sensory profile of raw honeys similar to pasteurized honeys can increase their consumption, which may have health-promoting value.

## Data availability statement

The raw data supporting the conclusions of this article will be made available by the authors, without undue reservation.

## Ethics statement

The studies involving humans were approved by Komisja Bioetyczna przy Śląskim Uniwersytecie Medycznym w Katowicach. The studies were conducted in accordance with the local legislation and institutional requirements. The participants provided their written informed consent to participate in this study. Written informed consent was obtained from the individual(s) for the publication of any potentially identifiable images or data included in this article.

## Author contributions

MK: Funding acquisition, Methodology, Project administration, Writing – review & editing. WS-B: Methodology, Resources, Writing – original draft. KS: Conceptualization, Data curation, Methodology, Writing – review & editing. LD: Fomal analysis, Software, Writing – review & editing. A-MS: Validation, Visualization, Writing – review & editing. AK: Investigation, Supervision, Writing – original draft. AB: Resources, Writing – original draft. JK: Writing – original draft.
